# Biopsychosocial Characteristics of Patients with Primary Fallopian Tube Carcinoma: Retrospective Single-Center Descriptive Pilot Study

**DOI:** 10.3390/jcm15020598

**Published:** 2026-01-12

**Authors:** Marcelina Migdał, Dorota Branecka-Woźniak, Joanna Błażejewska-Jaśkowiak, Edyta Skwirczyńska, Rafał Kurzawa

**Affiliations:** 1 Department of Gynecology and Reproductive Health, Faculty of Health Sciences, Pomeranian Medical University in Szczecin, 71-210 Szczecin, Poland; marcelina.migdal@pum.edu.pl (M.M.); dorota.branecka.wozniak@pum.edu.pl (D.B.-W.); rafal.kurzawa@pum.edu.pl (R.K.); 2 Medical Biotechnology and Laboratory Medicine, Department of the History of Medicine and Medical Ethics, Faculty of Pharmacy, Pomeranian Medical University in Szczecin, 72-210 Szczecin, Poland; edyta.skwirczynska@pum.edu.pl

**Keywords:** primary fallopian tube carcinoma, PFTC, psychosocial determinants, gynecologic oncology, preventive behaviors, psycho-oncology, women’s health

## Abstract

**Background:** Primary fallopian tube carcinoma (PFTC) is a rare gynecologic malignancy, and data describing its biopsychosocial characteristics remain limited. Understanding the biological, psychological, and social features of affected women may support patient-centered care and inform future research. **Methods:** This retrospective, single-center, descriptive pilot study included 20 patients with histopathologically confirmed PFTC treated in 2024–2025. Demographic, reproductive, clinical, preventive, and record-documented psychosocial variables were extracted from medical records. Categorical variables were analyzed using chi-square tests (or Fisher’s exact test where appropriate), and effect sizes were summarized using Cramér’s V. Proportions were reported with 95% confidence intervals using the Wilson method. **Results:** Half of the women were aged ≥70 years (50.0%; 95% confidence interval (CI): 29.9–70.1) and 65.0% had never been pregnant. Normal body mass index (BMI) predominated (65.0%). International Federation of Gynecology and Obstetrics (FIGO) stage was available for 12/20 patients; among those with documented staging, 58.3% were FIGO stage III. Preventive behaviors documented in medical records suggested suboptimal screening patterns: cervical cytology was classified as occasional in 75.0% of patients. Psycho-oncological support was documented in 45.0% of patients, and sleep problems were documented in 25.0%. An age-group difference in documented psycho-oncological support was observed (χ^2^ = 14.007; *p* = 0.007; Cramér’s V = 0.751); however, given the very small sample size and the distribution of observations across age categories, this finding should be interpreted as hypothesis-generating rather than confirmatory evidence. No association was observed between place of residence and FIGO stage in the subset with available staging data. **Conclusions:** In this small retrospective, single-center cohort, patients with PFTC were predominantly older and frequently nulligravid, while normal BMI was common. Record-documented psychosocial needs (including psycho-oncological support and sleep problems) were observed in a subset of patients and underscore the importance of systematic psychosocial assessment using validated tools in future studies and clinical pathways. Findings are preliminary and hypothesis-generating and support the need for larger prospective multicenter studies integrating comprehensive clinical and standardized psychosocial data in PFTC populations.

## 1. Introduction

Primary fallopian tube carcinoma (PFTC) is a rare gynecological malignancy, accounting for approximately 0.14–1.8% of all cancers of the female reproductive tract [1,2]. Despite the development of increasingly modern diagnostic methods, diagnosing primary fallopian tube carcinoma remains extremely challenging for clinicians. The difficulties encountered in daily clinical practice arise not only from the very low incidence of the disease but also from the marked heterogeneity and nonspecific nature of the symptoms [3,4,5]. In many cases, early manifestations overlap with other gynecologic conditions or present as vague abdominal complaints, significantly delaying the diagnostic process.

The tumor is typically diagnosed in postmenopausal women, with peak incidence between 60 and 70 years of age [6]. In postmenopausal women, gynecologic care may become more symptom-driven, with postmenopausal bleeding being a leading reason for consultation [7]. In addition, routine cervical cancer screening may be discontinued after the age of 65 if prior screening has been adequate, which may reduce routine preventive contact with gynecologic care [8,9]. Evidence on PFTC-specific risk factors remains limited; therefore, potential determinants are often discussed within the broader ovarian–fallopian tube–primary peritoneal cancer spectrum. Within this spectrum, reproductive history (including pregnancy-related factors) has been associated with risk patterns in gynecologic malignancies [10,11,12].

Nevertheless, due to the rarity of the disease and limitations of current epidemiological evidence, determining clear etiological pathways remains difficult. Recent pathological and molecular evidence increasingly supports the distal fallopian tube epithelium as a key site of origin for many pelvic high-grade serous carcinomas (HGSC), with serous tubal intraepithelial carcinoma (STIC) recognized as the earliest morphologically identifiable precursor lesion in a substantial proportion of cases [13,14]. This “tubal origin” model has important implications for prevention strategies, including opportunistic salpingectomy in average-risk women undergoing gynecologic or pelvic surgery and risk-reducing salpingo-oophorectomy in BRCA1/2 (breast cancer susceptibility genes) mutation carriers, although detailed clinical guidance for the detection and management of isolated STIC remains limited [3,15]. In parallel, clinicopathologic series highlight the clinical importance of accurate staging in PFTC and achieving optimal surgical outcomes in related high-grade serous disease [5].

Because PFTC predominantly affects postmenopausal women, the broader metabolic context is relevant to patient profiling in this setting. Menopause-related metabolic factors, including metabolic syndrome, have been investigated in relation to gynecologic cancers, including ovarian and fallopian tube cancers [16,17]. Against this biological and clinical background, characterizing patients with invasive primary fallopian tube carcinoma may provide additional insight into the spectrum of tubal pathology encountered in routine clinical practice.

Although previous scientific analyses have focused primarily on clinical variables, in recent years, increasing attention has been paid to the biopsychosocial aspects of cancer care. However, psychosocial data for PFTC are extremely limited due to the very low incidence of the disease and the predominance of clinicopathologic and outcome-focused publications [5,18]. In contrast, empirical evidence shows that women diagnosed with gynecologic malignancies frequently experience significant psychological distress, impaired quality of life, and unmet supportive care needs throughout the illness trajectory, underscoring the clinical and research relevance of psychosocial dimensions in gynecologic oncology [19]. Studies also emphasize that analyzing the emotional burden, coping mechanisms, health behaviors, and access to medical services is essential for improving patient outcomes [19,20]. Most available reports focus on histopathology, staging, and oncologic outcomes, with little or no information on patients’ psychological distress, coping strategies, health behaviors, or access to supportive care. As a result, clinicians must often extrapolate from studies conducted in patients with ovarian and other gynecologic cancers when considering the potential psychosocial needs of women with PFTC, even though the rarity and diagnostic complexity of this tumor may shape patients’ experiences in distinct ways.

Psychological distress is known to affect a substantial proportion of patients with gynecologic malignancies [19], and research indicates that women undergoing treatment for gynecologic cancers often experience fatigue, emotional tension, and barriers related to access to supportive services [20]. Furthermore, the COVID-19 pandemic led to a dynamic shift toward telemedicine, which improved access to care for selected groups of patients [21,22,23], although not all individuals benefited equally. Studies highlight that socioeconomic status influences health-seeking behaviors and access to specialized oncologic care [24]. At the same time, disparities between medical facilities and differences in the experience of clinical teams—particularly in the context of rare tumors—may also affect diagnostic and therapeutic pathways [25,26,27].

Understanding the epidemiological, clinical, reproductive, preventive, and psychosocial profile of patients with PFTC may help increase diagnostic vigilance among clinicians and support more individualized patient management. Given the diagnostic delays frequently described in the literature, there is a need to consider both biological and behavioral factors shaping access to care and symptom interpretation.

To the best of our knowledge, published data on the biopsychosocial profile of women diagnosed with primary fallopian tube carcinoma are extremely limited, and no study has provided a systematic characterization of these factors to date. Given the rarity of PFTC and the scarcity of disease-specific evidence, contextual comparisons in this manuscript occasionally draw on data from ovarian and other gynecologic cancers, which should be interpreted as indirect rather than PFTC-specific evidence.

Given these gaps, this retrospective single-center study aimed to characterize the biopsychosocial profile of patients diagnosed with primary fallopian tube carcinoma, including demographic, clinical, gynecologic, preventive, and psychological variables.

## 2. Materials and Methods

### 2.1. Study Design and Setting

We conducted a retrospective, single-center, descriptive pilot study of patients with histopathologically confirmed primary fallopian tube carcinoma (PFTC). Patients with an unclear primary tumor location or coexisting ovarian or peritoneal cancer, in whom the fallopian tube could not be unequivocally identified as the primary site, were excluded from the study. Data were collected at the Department of Operative Gynecology and Gynecologic Oncology of Adults and Adolescents at the Independent Public Clinical Hospital No. 2 in Szczecin in 2024–2025 (*n* = 20). All consecutive patients meeting the inclusion criteria within the analyzed period were included; no prior sample size estimation was performed due to the rarity of PFTC.

### 2.2. Data Sources and Extraction

Data were extracted from medical records, including medical history and progress notes. Psychosocial and lifestyle variables were derived exclusively from routine narrative clinical documentation; standardized, validated questionnaires were not part of routine assessment in this retrospective dataset. All non-staging variables were available for all patients in this cohort (20/20). Nevertheless, because psychosocial and lifestyle data were not collected using standardized instruments, the classification of these variables may be subject to documentation-related variability and potential misclassification.

### 2.3. Data Abstraction and Curation

Clinical, reproductive, preventive, and psychosocial variables were retrospectively extracted from patients’ medical records at our institution. Data extraction (chart review and abstraction) was performed by the study investigators. The extracted dataset was subsequently curated to ensure completeness and internal consistency across variables. Any unclear or ambiguous record entries were discussed among the investigators and resolved by consensus. Variables were coded as present only when explicitly documented in the medical record; no assumptions were made beyond recorded information.

### 2.4. Variables and Operational Definitions

Based on the medical records, data were collected for the following biopsychosocial variables:

- Age at diagnosis, categorized according to the ranges presented in the results table (e.g., 21–30, 41–50, 51–60, 61–70, 71+ years; detailed distributions are provided in Supplementary Table S1). Age categories were selected to reflect clinically meaningful life stages and to limit very small category sizes given the small sample.
- Reproductive status, categorized as never pregnant vs. ≥1 pregnancy in medical history.
- Body mass index (BMI) calculated from body weight and height, and categorized as underweight (<18.5 kg/m^2^), normal weight (18.5–24.9 kg/m^2^), overweight (25.0–29.9 kg/m^2^), and obesity class I (≥30.0 kg/m^2^).
- Place of residence, categorized as rural area, town <10,000 inhabitants, 10,000–50,000, 50,000–100,000, and ≥100,000 inhabitants.
- Clinical stage according to FIGO, recorded only when explicitly documented in the medical record. When operative and/or histopathological descriptions were incomplete and a precise FIGO stage could not be confirmed from the documentation, FIGO stage was coded as missing. FIGO stage was available for 12/20 patients (60%) and missing for 8/20 (40%). No imputation was performed. Analyses involving FIGO stage were restricted to cases with available staging information (*n* = 12).
- Living conditions, recorded in the medical records as average, good, or very good.
- Cervical cytology screening history, categorized as follows:
  (I) Regular (guideline-adherent): the medical record indicated that cytology was performed regularly and/or according to recommended screening intervals (e.g., explicit documentation of regularity and/or screening intervals);
  (II) Occasional/irregular (non-adherent): cytology was documented as sporadic, irregular, or rare (i.e., not performed at guideline-recommended intervals);
  (III) Never: the medical record indicated that cytology had never been performed.

Classification relied on the treating clinician’s record-documented assessment of screening regularity; however, individual test dates and original reports were not consistently available in the records accessible for this study, precluding independent verification of screening intervals.

- Substance use, categorized as none, tobacco smoking, or alcohol consumption (categories not mutually exclusive) based on medical record documentation,
- Use of psycho-oncological support was coded as present when a psycho-oncological service was recorded as performed in the hospital information system/medical record (i.e., an entry indicating a completed psycho-oncology procedure/encounter). The timing relative to diagnosis or treatment was not consistently documented and therefore was not analyzed.
- Sleep status, documented as no sleep problems, difficulty initiating sleep, or insomnia. For descriptive reporting and analysis, difficulty initiating sleep and insomnia were combined into a single category (“sleep problems”) and contrasted with no sleep problems. Sleep status was documented for all patients in this cohort.
- BRCA mutation status and other germline genetic data were not systematically recorded in the available medical documentation and were therefore not included in the present analysis.

### 2.5. Statistical Analysis

Statistical analyses were conducted using IBM SPSS Statistics 26.0 (IBM Corp., Armonk, NY, USA). Categorical variables were compared using the chi-square test of independence (or Fisher’s exact test or the Monte Carlo procedure when expected cell counts were low). Where applicable, Cramér’s V was reported as an effect size measure. Proportions are presented with 95% confidence intervals calculated using the Wilson method. A two-sided significance level of *p* < 0.05 was adopted. Due to the exploratory nature of the analyses and the small sample size, no corrections for multiple comparisons were applied. Missing data concerned only the FIGO stage, as described above; all other variables were available for all patients in this cohort. However, psychosocial and lifestyle variables were derived from routine narrative clinical documentation without standardized instruments and may therefore be subject to documentation-related variability and potential misclassification.

### 2.6. Ethics

The study received a positive opinion from the Local Bioethics Committee (protocol number: RPW/9510/2022P; date of approval: 17 October 2022). Approval for the review and analysis of anonymized medical records was granted by the department management. Due to the retrospective design and the use of anonymized documentation, individual patient consent was not required.

## 3. Results

Unless otherwise specified, proportions are reported for the full cohort (*n* = 20); analyses involving FIGO stage are reported for staged cases only (*n* = 12).

### 3.1. Biological Profile

A total of 20 patients with a diagnosis of PFTC were included in the analysis. The age distribution was shifted toward older categories, with 50.0% of patients aged ≥70 years (10/20; 95% CI: 29.9–70.1). Regarding reproductive history, 65.0% of women had never been pregnant (13/20; 95% CI: 43.3–81.9). Normal body weight predominated (65.0%; 13/20; 95% CI: 43.3–81.9).

FIGO stage was available for 12/20 patients (60%) and missing for 8/20 (40%). Among staged cases (*n* = 12), FIGO stage III was the most frequent stage at diagnosis (58.3%; 7/12; 95% CI: 32.0–80.7), followed by stage I (33.3%; 4/12) and stage II (8.3%; 1/12) (Table 1).

Detailed distributions of age and anthropometric categories are provided in the Supplementary Materials.

### 3.2. Psychological Profile

The medical documentation indicated the use of psycho-oncological support in 45% of patients (9/20; 95% CI: 25.8–65.8). Sleep problems (difficulty falling asleep or insomnia) were documented in 25% (5/20; 95% CI: 11.2–46.9) (Table 2).

### 3.3. Social Aspects

The largest proportion of patients lived in cities with ≥100,000 inhabitants (45.0%; 95% CI: 25.8–65.8). A detailed distribution of social and lifestyle variables is presented in Supplementary Table S2. Regarding preventive behaviors, occasional cervical cytology testing was predominant: 75% (15/20; 95% CI: 53.1–88.8), while only 20% (4/20; 95% CI: 8.1–41.6) was classified as regular screening. The uneven distribution of these categories was confirmed by the chi-square test: χ^2^ = 16.300; df = 2; *p* < 0.001. Most patients had living conditions documented as good or very good (17/20; 85%; 95% CI: 64.0–94.8). Tobacco smoking was documented in 30.0% (6/20; 95% CI: 14.5–51.9) and alcohol consumption in 5.0% (1/20; 95% CI: 0.9–23.6). Given the retrospective, record-based nature of these variables and the potential for documentation/underreporting bias, these values should be interpreted with caution (Table 3).

### 3.4. Key Associations

Table 4 summarizes exploratory chi-square analyses for selected pairs of categorical variables. Given the small sample size and small, uneven cell counts in several cross-tabulations, these results should be interpreted as hypothesis-generating; no corrections for multiple comparisons were applied.

An age-group difference in documented psycho-oncological support was observed (χ^2^ = 14.007; df = 4; *p* = 0.007; Cramér’s V = 0.751). However, several age categories contained very small or zero cell counts (Table 5), and the estimate may therefore be unstable; this finding should not be interpreted as confirmatory evidence of a robust association.

No association was observed between place of residence and psycho-oncological support (χ^2^ = 1.776; df = 2; *p* = 0.411; Cramér’s V = 0.30). A chi-square goodness-of-fit test showed an uneven distribution across the three cervical cytology categories (χ^2^ = 16.300; df = 2; *p* < 0.001), with “occasional” screening being dominant; this result is descriptive for this cohort and does not constitute a population-level risk assessment. No association was found between place of residence and FIGO stage among staged cases (χ^2^ = 6.395; df = 8; *p* = 0.603; FIGO available for *n*= 12).

Table 5 provides the underlying cross-tabulation and shows that several age strata contain very small counts, supporting cautious interpretation of the observed association.

## 4. Discussion

This discussion should be interpreted in light of several limitations: the small sample size (reflecting the rarity of PFTC), missing staging data for 8 of 20 patients, and reliance on narrative medical records rather than standardized instruments for psychosocial and lifestyle variables. Accordingly, the analyses are exploratory and underpowered, and the observed associations should be considered descriptive and hypothesis-generating rather than confirmatory. With these constraints in mind, we first contextualize the clinical profile and pathways to presentation in our cohort, and then discuss the psychosocial dimension and its implications for supportive care in this understudied population. We also lacked systematic information on BRCA mutation status and STIC lesions, which precluded analyses of genetic risk or precursor pathology in relation to the biopsychosocial profile. Moreover, PFTC-specific psychosocial literature remains scarce; therefore, comparisons necessarily rely on data from other gynecologic malignancies.

In this cohort, the age profile of patients with primary fallopian tube carcinoma was shifted toward the eighth decade of life (half of women were ≥70 years old; Table 1). This distribution differs from that reported by other researchers, who most commonly indicate the mean age of affected patients as falling within the 5th–6th decade, with a median of approximately 55–58 years [5,6]. These differences may result from local demographics or diagnostic pathways and therefore remain an observation requiring further exploration. Epidemiological analyses estimate that primary fallopian tube carcinoma represents only approximately 0.14–1.8% of female genital tract malignancies, with an incidence of 3–4 cases per million women per year, which significantly limits the availability of large-scale comparative studies and may partly explain divergences between published cohorts. The rarity of PFTC also contributes to a general absence of population-level clinical pathways and diagnostic vigilance typical for more common gynecologic cancers [2]. One possible explanation is that the older age of patients reflects less frequent contact with gynecologists due to menopause having occurred many years earlier and a reduced perceived need for routine gynecologic visits, which could theoretically contribute to delayed recognition of nonspecific symptoms. Given the retrospective, descriptive design, this hypothesis cannot be tested and causal inferences cannot be made; therefore, interpretations of behavioral pathways remain speculative.

After menopause, gynecologic care often becomes more symptom-driven, which may reduce opportunities for routine preventive contact and delay assessment of nonspecific symptoms. In this context, our descriptive findings in this small cohort are broadly consistent with a pattern of predominantly non-preventive care-seeking behavior [7,8,9].

In the studied group, “occasional” cervical cytology was the dominant pattern, which suggests sporadic contact with the healthcare system. This finding should not be interpreted as indicating an increased risk of PFTC; rather, it may reflect underutilization of opportunities for preventive care and timely symptom assessment in this age group. In this context, regular gynecologic visits may facilitate earlier recognition of nonspecific complaints or abnormal findings during examination and should be viewed as an organizational element of care rather than a specific preventive measure for fallopian tube carcinoma. Beyond patterns of healthcare utilization, our cohort also showed a distinct reproductive profile, which is frequently discussed within the broader ovarian–tubal–peritoneal cancer spectrum.

The high proportion of patients who had never been pregnant (Table 1), in the absence of a control group, does not allow for a definitive determination of the impact of nulliparity on PFTC risk. However, the finding is directionally consistent with data indicating a protective role of successive pregnancies against cancers of the ovary–fallopian tube–peritoneum spectrum, with the strongest risk reduction occurring after the first three births [10,11]. These associations are thought to be driven by biological mechanisms, including a reduced number of ovulations and hormonal changes in parous women. In practice, this underscores the importance of obstetric history in patients with suspected PFTC; however, according to recent meta-analyses, quantitative data specifically on fallopian tube cancer remain limited [12]. These observations should therefore be interpreted as contributing to clinical risk profiling rather than as a basis for causal conclusions about PFTC.

In our cohort, most patients had a normal body weight (Table 1). This finding aligns with results of other authors, who did not document a clear and independent role of metabolic profile in the risk of ovarian and fallopian tube cancers [16], in contrast with endometrial cancer [17]. This result is descriptive and indicates that PFTC can occur in women without significant metabolic comorbidities. Among patients with documented FIGO stage, the most frequent category was stage III (58.3%, Table 1). This pattern is directionally consistent with reports that PFTC often presents at an advanced stage and may be diagnosed late due to nonspecific or absent early symptoms [5], although our finding should be interpreted cautiously given the small number of staged cases. The lack of validated screening tools or early detection strategies for fallopian tube carcinoma further contributes to the pattern of predominantly advanced-stage diagnosis. This gap in early detection is consistent with current STIC-focused literature, which indicates that precursor tubal lesions are often detected only incidentally and that evidence-based pathways for identification and management remain under development [3]. Reviews of fallopian tube carcinoma similarly describe that most patients present with advanced-stage disease at diagnosis, reflecting the absence of effective screening strategies and the nonspecific nature of early symptoms [1].

The psychological and social dimensions—rarely addressed in PFTC literature—complement the clinical profile observed in our cohort. Nearly half of the patients had documented psycho-oncological support, and one in four reported sleep disturbances (Table 2), indicating that psychosocial aspects are relevant even in this small and clinically heterogeneous group. Oncologic literature emphasizes the importance of assessing sleep problems, distress, and depressive symptoms among patients with cancer, as well as the need for psycho-oncological support regardless of disease stage [19,20]. Data specific to PFTC are lacking; however, poor sleep quality is common among patients with gynecologic cancers and has been linked to reduced quality of life, supporting the need for routine evaluation of sleep disturbances in clinical care [28]. In our study, the highest proportion of patients with documented psycho-oncological support was in the 61–70 year age group (Table 4). This pattern may reflect age-related differences in symptom interpretation, help-seeking behavior, or referral practices, but such mechanisms cannot be inferred from retrospective record-based data and remain speculative. Alternative explanations cannot be excluded, such as organizational factors influencing referral patterns or documentation practices at the study center, which might also contribute to the relatively high proportion of patients with recorded psycho-oncological support in this cohort. Information about available support and routine inquiries about emotional well-being and sleep may therefore be warranted in this context and may be worth considering irrespective of PFTC stage, although our observations remain descriptive and hypothesis-generating. In the present study, these aspects were captured only through narrative documentation rather than standardized psychometric tools, which underscores both the presence of psychosocial needs and the methodological gap that should be addressed in future PFTC research.

The lack of a statistically significant association between place of residence and use of psycho-oncological support (Table 4), alongside descriptively lower proportions among rural patients, should be interpreted cautiously and viewed as a hypothesis-generating observation rather than evidence of a robust effect. It suggests that actual access to specialist support may be influenced not only by geographical factors but also by organizational arrangements and how services are offered. Recent reports highlight the role of simple remote-support options, such as video consultations or same-day telephone check-ins [21,22]. Clear information about such accessible forms of contact may reduce barriers related to travel, time, or cost an especially important consideration for older patients, among whom telephone contact often remains a more acceptable option than video consultations [23]. Allowing patients, particularly older adults, to choose their preferred form of remote communication may help reduce geographic barriers observed in peripheral populations.

In our cohort, no association was observed between place of residence and FIGO stage (Table 4). This finding suggests that delayed diagnosis of PFTC may be more strongly related to nonspecific symptoms than to geographical location, although it does not exclude the possibility that territorial barriers may affect other aspects of care.

In the studied group, most patients described their living conditions as good or very good (Table 3). The literature indicates that low socioeconomic status is associated with disparities in cancer prevention, access to diagnostic services, and treatment outcomes [24], a pattern also observed in gynecologic cancers [25]. The descriptive result in our sample indicates that, in this particular setting, PFTC was a diagnosed in women reporting relatively stable and favorable socioeconomic conditions, and therefore should not be viewed as restricted to populations with clearly adverse socioeconomic status.

Exposure to substances such as tobacco and alcohol was observed in 30% and 5% of patients, respectively (Table 3). Given the small sample size and the potential underreporting inherent to medical documentation, these values provide only a preliminary depiction of the group and cannot be generalized. Nevertheless, available reviews on primary fallopian tube carcinoma also indicate that data on the impact of smoking and other lifestyle factors are sparse and inconsistent, with many conclusions extrapolated from ovarian and peritoneal cancer research [26,27].

Overall, this descriptive pilot study highlights clinically relevant patterns and the presence of documented psychosocial needs in an understudied PFTC population. These observations should be interpreted as hypothesis-generating and may inform the design of future adequately powered, multicenter research incorporating standardized psychosocial assessment alongside clinical data.

### Future Directions

Given the rarity of PFTC and the small, single-center nature of our cohort, future research should focus on multicenter registries and prospective studies that can provide adequately powered, disease-specific data. Such projects should integrate detailed clinical and pathological information with standardized assessments of psychosocial functioning, sleep, and quality of life, rather than relying solely on narrative chart documentation. In addition, refining preoperative diagnostic pathways and risk stratification tools for suspected tubal malignancies may help clarify whether the patterns observed in this cohort reflect local variability or more generalizable features of PFTC.

## 5. Conclusions

1. In this analyzed single-center series of PFTC cases, diagnoses predominantly occurred in older women, with a high proportion of nulligravidity and predominantly normal BMI. This pattern is broadly consistent with the existing literature; however, given the small sample size, these observations remain descriptive and hypothesis-generating.
2. The observed use of psycho-oncological support and reported sleep disturbances indicate the presence of psychosocial needs among patients with PFTC and may support consideration of brief, routine assessments of psychological well-being in clinical practice for this group, to be confirmed in larger cohorts.
3. Given the retrospective, single-center design and the small sample size, the presented findings are hypothesis-generating rather than confirmatory. None of the observations in this study should be interpreted as causal or practice-changing; instead, they provide preliminary signals that support the need for adequately powered, multicenter analyses that systematically integrate both clinical and psychosocial data among patients with PFTC.

## Figures and Tables

**Table 1:** Biological and clinical profile; *n* = 20; FIGO available for *n* = 12 and unavailable for *n* = 8.

Variable	Category	*n*	%	95% CI
Age	≥70 years	10	50.0	29.9–70.1
Reproductive status	Never pregnant	13	65.0	43.3–81.9
BMI	Normal weight	13	65.0	43.3–81.9
FIGO clinical stage *	I	4	33.3	13.8–60.9
	II	1	8.3	1.5–35.4
	III	7	58.3	32.0–80.7

* FIGO calculations based on data from 12 patients. BMI—body mass index; FIGO—International Federation of Gynecology and Obstetrics; CI—confidence interval.

**Table 2:** Psychological profile; *n* = 20.

Variable	Category	*n*	%	95% CI
Psycho-oncological support	Yes	9	45.0	25.8–65.8
Sleep	Difficulties/insomnia	5	25.0	11.2–46.9

**Table 3:** Social profile; *n* = 20.

Variable	Category	*n*	%	95% CI
Place of residence	City ≥ 100,000	9	45.0	25.8–65.8
Cytology	Regular	4	20.0	8.1–41.6
	Occasional	15	75.0	53.1–88.8
	Never	1	5.0	0.9–23.6
Substances	Nicotine	6	30.0	14.5–51.9
	Alcohol	1	5.0	0.9–23.6
Living conditions	Good/very good	17	85.0	64.0–94.8

**Table 4:** Associations between variables.

Variable Pair	Test	χ^2^	df	*p*	Cramér’s V	Notes
Psycho-oncological support × age (categories)	χ^2^	14.007	4	0.007	0.751	Exploratory; small and uneven cell counts across age categories; interpret cautiously; see Table 5
Place of residence × psycho-oncological support	χ^2^	1.776	2	0.411	0.30	Exploratory; not statistically significant. Any apparent differences in proportions should be interpreted cautiously due to small cell counts.
Cervical cytology (3 categories)—distribution	χ^2^	16.300	2	<0.001	-	Uneven distribution; dominance of “occasional” screening.
Place of residence × FIGO stage (I–III)	χ^2^	6.395	8	0.603	-	Exploratory; FIGO available for *n* = 12 patients only.

χ^2^—chi-square statistic; df—degrees of freedom; *p*—significance level; Cramér’s V—effect size (reported where applicable). The “Cervical cytology—distribution” row refers to a one-dimensional goodness-of-fit test; Cramér’s V is not applicable. All analyses are exploratory due to the small sample size; no correction for multiple comparisons was applied.

**Table 5:** Cross-tabulation of age group and documented psycho-oncological support (*n* = 20).

Age (Years)	Psycho-Oncological Support: Yes *n* (%)	Psycho-Oncological Support: No *n* (%)	Total (*n*)
21–30	1 (100.0)	0 (0.0)	1
41–50	1 (50.0)	1 (50.0)	2
51–60	0 (0.0)	5 (100.0)	5
61–70	6 (85.7)	1 (14.3)	7
71+	1 (20.0)	4 (80.0)	5
Total	9 (45.0)	11 (55.0)	20

Statistical test: chi-square test for independence (χ^2^ = 14.007; df = 4; *p* = 0.007; Cramér’s V = 0.751).

## Data Availability

The data supporting the findings of this study are available from the corresponding author upon reasonable request. Due to patient privacy restrictions, raw medical records cannot be publicly shared.

## Supplementary Materials

The following supporting information can be downloaded at https://www.mdpi.com/article/10.3390/jcm15020598/s1. Table S1: Detailed biological characteristics of patients with primary fallopian tube carcinoma (PFTC) (*n* = 20); Table S2: Detailed social, psychological and lifestyle characteristics (*n* = 20).

## Abbreviations

The following abbreviations are used in this manuscript:

PFTC	Primary Fallopian Tube Carcinoma
HGSC	High-Grade Serous Carcinoma
FIGO	International Federation of Gynecology and Obstetrics staging system
BMI	Body Mass Index
CI	Confidence Interval
SES	Socioeconomic Status
SPSS	Statistical Package for the Social Sciences
df	Degrees of Freedom
χ^2^	Chi-square test statistic
