# Supplementary material for: Biopsychosocial Characteristics of Patients with Primary Fallopian Tube Carcinoma: Retrospective Single-Center Descriptive Pilot Study
Source: J Clin Med. 2026 Jan 12;15(2):598. doi: 10.3390/jcm15020598 (PMC12842301; doi:10.3390/jcm15020598)
Supplement: Supplementary file 1 [file jcm-15-00598-s001.zip › jcm-4059641-SI.pdf]

Supplementary Materials: Detailed Biopsychosocial Characteristics of Patients with Primary Fallopian Tube Carcinoma (PFTC)

Table S1. Detailed biological characteristics of patients with primary fallopian tube carcinoma (PFTC) (n = 20).

Variable	Category	n	%	95% CI
Age (years)	21–30	1	5.0	0.9–23.6
	31- 40	0	0	0.0–16.1
	41–50	1	5.0	0.9–23.6
	51–60	3	15.0	5.2–36.0
	61–70	5	25.0	11.2–46.9
	>70	10	50.0	29.9–70.1
Reproductive status	Never pregnant	13	65.0	43.3–81.9
	≥1 pregnancy	7	35.0	18.1–56.7
BMI	Underweight	1	5.0	0.9–23.6
	Optimal weight	13	65.0	43.3–81.9
	Overweight	5	25.0	11.2–46.9
	Obesity class I	1	5.0	0.9–23.6

Table S2. Detailed social, psychological and lifestyle characteristics (n = 20).

Variable	Category	n	%	95% CI
Place of residence	Rural area (village)	5	25.0	11.2–46.9
	Town <10,000 inhabitants	1	5.0	0.9–23.6
	Town 10,000–50,000 inhabitants	2	10.0	2.8–30.1
	City 50,000–100,000 inhabitants	3	15.0	5.2–36.0
	City >100,000 inhabitants	9	45.0	25.8–65.8
Living conditions	Average	3	15.0	5.2–36.0
	Good	16	80.0	58.4–91.9
	Very good	1	5.0	0.9–23.6

Cervical cytology	Regularly	4	20.0	8.1–41.6
	Occasionally	15	75.0	53.1–88.8
	Never	1	5.0	0.9–23.6
Substance use	None	13	65.0	43.3–81.9
	Nicotine	6	30.0	14.5–52.0
	Alcohol	1	5.0	0.9–23.6
Psycho-oncological support	Yes	9	45.0	25.8–65.8
	No	11	55.0	34.2–74.2
Sleep	Normal	15	75.0	53.1–88.8
	Difficulty falling asleep	4	20.0	8.1–41.6
	Insomnia	1	5.0	0.9–23.6
